# On the occasion of world kidney day 2017; obesity and its relationship with chronic kidney disease

**DOI:** 10.15171/jnp.2017.18

**Published:** 2017-01-05

**Authors:** Leila Mahmoodnia, Mohammad Reza Tamadon

**Affiliations:** ^1^Department of Internal Medicine, Shahrekord University of Medical Sciences, Shahrekord, Iran; ^2^Department of Internal Medicine, Semnan University of Medical Science, Semnan, Iran

**Keywords:** Chronic kidney disease, Obesity, World kidney day, End-stage renal disease

## Abstract

**Context:**

Numerous studies have reported the impact of obesity in the incidence of chronic kidney disease (CKD). Some studies have suggested the direct role of obesity in the incidence of CKD, while some other studies suggest an indirect effect caused by the effects of obesity on blood pressure and diabetes.

**Evidence Acquisition:**

PubMed, EBSCO, Web of Science, directory of open access journals (DOAJ), EMBASE, and Google Scholar have been searched.

**Results:**

Recent studies have presented more strong evidences on the role of obesity on the incidence of CKD. The double role of obesity in the incidence of CKD has also been mentioned in some studies.

**Conclusions:**

Such an additional effect arises from the impact of obesity on the incidence of some conditions and diseases such as cardiovascular disease, hypertension, and diabetes, which in turn are involved in the incidence of CKD and are considered as its risk factors.

**Implication for health policy/practice/research/medical education:**

Prevention is always better than a cure. Following a healthy lifestyle can protect us against many diseases; it is demonstrated in the slogan of the World Kidney Day suggesting a healthy lifestyle for a healthy kidney.

## 1. Context

Obesity is one of the problems facing the healthcare system in the world. According to the reports by the World Health Organization (WHO), in 2008 around 1.4 billion were overweight and 500 million were obese (1). Two-thirds of adults in the United States are overweight and about one-third of them are obese (2).

Obesity is a complex problem which can play a role in the incidence of many metabolic problems such as diabetes, dyslipidemia, hypertension, and metabolic syndrome. Recently, various studies have reported its role in chronic kidney disease (CKD) as well (3,4). Some studies suggest that obesity may increase the risk of developing CKD by as much as two to three times (3).

Obesity is associated with increased glomerular filtration, increased risk of cardiovascular diseases and microalbuminuria (5-10). Some studies have suggested that kidney dysfunction in obese people is an independent risk factor for developing CKD (11,12).

A study suggests that the incidence of abdominal obesity in young people is an independent risk factor for the incidence of CKD and is associated with the race. Therefore, obese young people are recommended to conduct kidney function tests on a regular basis (13).

Given the importance of CKD, the increasing incidence of obesity, differences in the living conditions of people in different communities (14,15), and the likely relationship between obesity and CKD, this study aimed to review the previously published papers to evaluate the relationship between these two (obesity and CKD) and determine the related prevention methods.

## 2. Evidence Acquisition

PubMed, EBSCO, directory of Open Access Journals (DOAJ), Google Scholar, and Web of Science were searched with key words as chronic kidney disease, end-stage renal disease and obesity. We searched and reviewed the papers published in scientific journals during a 10-year period (from 2007 to 2016).

## 3. CKD and its risk factors

CKD is a health problem that can progress and lead to end-stage renal disease (ESRD). It can increase mortality from heart diseases. Available statistics indicate that 10% to 16% of adults in different parts of the world are affected by CKD (16).

Several underlying items and risk factors for developing CKD have been proposed, among which we may note the followings: genetic factors, age, sex, race, culture, family history, drug use, smoking, socioeconomic status, and concomitant disorders such as hypertension and diabetes.

Some studies have suggested the influence of genetic factors and environmental factors in the development of CKD (17). Such studies have noted the relationship between the gene associated with glomerular filtration rate and the disease, they suggest that mutations in this gene can be associated with changes in renal function (18). Apolipoprotein L1 (*APOL1*) gene is the other gene associated with CKD; it increases the risk of nephropathy induced by immunodeficiency syndrome, focal glomerulosclerosis, CKD associated with hypertension, and CKD unrelated to diabetes especially in the black population (17). In addition, the gene associated with the renin angiotensin system also plays a role in the incidence of CKD (19).

In 23% of dialysis patients, there is a family history of ESRD. The previously conducted studies have recommended performing kidney disease tests and examinations for all the family members of dialysis patients (20).

In previous studies, ESRD was more prevalent in males. In other words, it has been 1.41 time more prevalent among males than among females (21).

People’s race is the other important risk factors for ESRD. It is well-documented that African-Americans have a higher prevalence of ESRD than Americans European people. The risk of the incidence of ESRD in black women was higher than that in black men. On contrary, the risk of incidence of ESRD was lower in white women, as compared with the white men (22).

Age was also a factor influencing the incidence of CKD. In people over 30 years of age, with increasing every 10 years of age, the risk of CKD increases by 1.45 to 2.18 times (23). Socioeconomic status also plays a role in the incidence of CKD. According to previously conducted studies, the risk of developing CKD in people with low socioeconomic status increased by up to 2.4 times (24).

Smoking also plays a role in the incidence of CKD. Smoking is associated with increased incidence of pro-inflammatory state, oxidative stress, endothelial dysfunction, glomerulosclerosis, and tubular atrophy and can play a role in the increased risk of CKD (25).

Among the other known causes of chronic renal failure, we may note the followings entities as nephrotoxic drugs (26), acute renal damage (27), diabetes mellitus (22), hypertension (28), obstructive sleep apnea (OSA) (25), elevated heart rate (29), periodontal diseases (30), and finally obesity (31).

Obesity is one of the most important risk factors for the incidence of CKD which is preventable (31). Glomerular hypertrophy and increased filtration can accelerate kidney damage through increasing the pressure of glomerular capillary wall and reducing the density of cells in the Bowman’s capsule (31).

Previously conducted studies have investigated the role of obesity in the incidence of CKD. In a study which was conducted in Sweden, people aged 18 to 74 years old with a creatinine level of 3.4 in males and 2.8 in females were investigated. The results of the mentioned study showed that the risk of developing CKD in people aged over 20 years with a body mass index (BMI) higher than 25 was up to 3 times more than the risk in those with a BMI less than 25. Obesity in men (BMI more than 30) and excessive obesity in women (BMI more than 35) can increase the risk of CKD by three to four times (25).

Obesity is an epidemic in the 21st century. Obesity is one of the risk factors for diabetes type II, cancer, hypertension, dyslipidemia, cardiovascular diseases, sleep apnea, and CKD. Obesity increases the risk of hypertension, atherosclerosis, and diabetes type II, which in turn increase the risk of developing CKD (32).

Obesity and overweight, with or without metabolic syndrome, are associated with an increased risk of CKD. Studies have shown that overweight and obesity, even in the absence of metabolic syndrome, are not benign factors and can result in developing CKD. Obesity and overweight are the risk factors for CKD, however, this relationship is not very strong. Overall, the previously conducted studies recommended conducting more studies to prove the relationship (33).

Obesity also has a direct effect on the incidence of CKD. According to the results of a study, a 12% body weight loss in patients with advanced diabetic nephropathy in the short term improved the glomerular filtration and kidney function indicators and reduced the risk factors for kidney disease progression (34).

Early diagnosis of CKD risk factors makes it possible to eliminate them and prevent the progress of the disease. Obesity is one of the preventable risk factors. According to a study, the risk of CKD in the obese patients is 1.71 times more than that in the general population (odds ratio: 1.71, 95% CI: 1.14-2.59). The mentioned study noted that many CKD risk factors including obesity can be detected and corrected before CKD diagnosis, therefore early detection and correction of these factors is of great importance (35).

In another study it was observed that obese individuals with CKD differed from obese people without CKD in terms of behavioral factors, lifestyle, and attitudes toward obesity. It was also found that the studied people had inadequate knowledge on the role of obesity in the incidence of CKD. However, the study suggests conducting further studies to prove the role of obesity in the incidence of CKD (36).

Other forms of obesity, such as abdominal obesity, are among the risk factors for CKD. Abdominal obesity can be an independent risk factor for the development of albuminuria, even in people with normal blood pressure and normal glucose level (13). Accordingly, it highlights the value of adopting measures to prevent abdominal obesity, especially in young people.

Obesity is known as one of the risk factors for CKD, but its role has not clearly been determined yet. Previous studies have suggested the role of changes in the secretion of adipokine, however further studies are needed to prove its role (37).

Numerous other studies have reported the impact of obesity in the incidence of CKD. Some studies have suggested the direct role of obesity in the incidence of CKD while some other studies suggest an indirect effect caused by the effects of obesity on blood pressure and diabetes (38-40). However, more recent studies have presented more strong evidences on the role of obesity on the incidence of CKD (41,42).

The double role of obesity in the incidence of CKD has also been mentioned in some studies. Such an additional effect arises from the impact of obesity on the incidence of some conditions and diseases such as cardiovascular disease, hypertension, and diabetes, which in turn are involved in the incidence of CKD and are considered as its risk factors (43,44).

## 4. Conclusions

Many people around the world are suffering from CKD. Sometimes the disease is latent and asymptomatic, thus the patient is not aware of its own condition. In such a condition, the patient becomes aware of the disease when it has progressed much and even reached an irreversible point. Early diagnosis of the disease and the identification of its risk factors and correcting them is of great value.

Obesity (including abdominal obesity) is one of the risk factors for CKD. Obesity is associated with other chronic diseases such as cardiovascular diseases, diabetes, and hypertension which are among the predisposing factors for the development of CKD.

Obesity is a modifiable risk factor and it can be prevented through the utilization of a healthy diet, exercise, and physical activity.

Given the importance of early diagnosis of CKD and its role in the prevention of irreversible kidney damages, it is better to consider renal function tests as part of routine examinations for all high-risk groups including those who are obese or overweight. It can help to modify the risk factors in a timely manner and prevent the progress of the disease and the subsequent irreversible complications.

Prevention is always better than a cure. Following a healthy lifestyle can protect us against many diseases; it is demonstrated in the slogan of the World Kidney Day suggesting a healthy lifestyle for a healthy kidney.

## Author’s contribution

LM and MRT wrote the paper equally.

## Conflicts of interest

There was not conflict of interest to declare.

## Funding/Support

None.
